# The effect of red-fleshed pitaya ( *Hylocereus polyrhizus*) on heat shock protein 70 and cortisol expression in strenuous exercise induced rats

**DOI:** 10.12688/f1000research.17533.1

**Published:** 2019-01-30

**Authors:** Novita Sari Harahap, Aznan Lelo, Ambrosius Purba, Awaluddin Sibuea, Rina Amelia, Zulaini Zulaini

**Affiliations:** 1 Department of Sports Sciences, Faculty of Sports Sciences, Universitas Negeri Medan, Medan, North Sumatra, 20221, Indonesia; 2 Department of Pharmacology, Faculty of Medicine, University of Sumatra Utara, Medan, North Sumatra, 20155, Indonesia; 3 Department of Physiology, Faculty of Medicine, Padjajaran University, Bandung, West Java, 45363, Indonesia; 4 Surgery Division, Dr. T. Mansyur Tanjung Balai Hospital, Tanjungbalai, North Sumatra, 21312, Indonesia; 5 Department of Public Health, Faculty of Medicine, University of Sumatra Utara, Medan, North Sumatra, 20155, Indonesia

**Keywords:** Red-fleshed pitaya, HSP70, cortisol, strenuous exercise

## Abstract

**Background: **Oxidative stress from exercise can contribute to damaging cells, increasing heat shock protein 70 (HSP70) and suppressing the immune system in the body. This research aimed to determine the antioxidant potential of red-fleshed pitaya extract on HSP70 and cortisol expression in rats which were subjected to strenuous exercise.

**Methods: **The subjects of this research were 32 Sprague Dawley male rats, aged 3 months, with an average weight of 200 g. Red-fleshed pitaya extract was obtained from methanol extraction process; a maceration technique was performed and the extract was concentrated using an air-drying method. Rats were randomly divided into four groups. Group 1 were subjected to strenuous exercise and treated with distilled water only; while Groups 2, 3 and 4 were subjected to strenuous exercise and treated with 100 mg/kg body weight, 200 mg/kg body weight and 300 mg/kg body weight of red-fleshed pitaya extract, respectively. Strenuous exercises in rats was performed by intense swimming of 20 min/day, 3 days a week for 3 weeks. HSP70 expression and cortisol were measured with Enzyme-Linked Immune Sorbent Assay (ELISA) method.

**Results: **There was a significant reduction of HSP70 (p=0.000) and cortisol expression (p=0.000) between the groups. Also, there was a significant difference in the average decreasing of HSP70 expression between group 4 and either groups 1 or 2 (p=0.000). However, a significant difference between groups 4 and 3 was not observed (p=0.813). Lastly, a significant difference was found in the average decrease of cortisol expression between groups 4 and 1 (p=0.000), 2 (p=0.000), and 3 (p=0.000) respectively.

**Conclusion: **Red-fleshed pitaya is potential to be utilized as antioxidant to decrease the HSP70 and cortisol expression.

## Introduction

Physical activity is an activity which has various influences and significant effects on the body. The effect of regular physical activity is a positive influence on biological functions, and will improve health and the antioxidant defense system in order to protect body from the negative effects of oxidative damage ^1^. Strenuous exercise tends to trigger free radical compound production. Moreover, this impairs the balance of free radicals and antioxidants as a result of oxidative stress ^2– 4^. Research has discovered that oxidative stress from strenuous exercise reduces performance as it damages cells ^5^, causing pain and muscles fatigues ^6^, lowering antioxidant levels ^7, 8^, increasing the expression of heat shock protein 70 (HSP70) ^9, 10^ and suppressing the immune system ^11^.

During an intense workout, self-defense and self-adaptation depend on the body condition which can be observed from HSP70 protein expression. Increases of HSP70 in muscles indicates a response to protect muscles cells from oxidative stress. HSP70 expression is an adaptation mechanism and a sign of damaging cells caused by oxidative stress ^12^. Previous research reported that workouts increase HSP70 expression ^10^. Strenuous exercise is a physical stressor in the body and as a result, adrenocorticotropic hormone(ACTH) is secreted by hypothalamus hypo-physis anterior and triggers the adrenal cortex to produce cortisol ^13^. The escalation of cortisol is influenced by the intensity and duration of training that leads to a suppression of the immune system, resulting in a decline of antibody. Cortisol can be a sign that the body encounters a decline in the immune system due to heavy training ^14^. Antioxidants can detoxify the lipid peroxide produced during exercise, which can eliminate radicals and reduce the inflammatory response to exercise. Therefore, it can prevent a muscle damage from exercise ^15^.

The body needs exogenous antioxidants to neutralize and prevent chain reactions from free radicals formed from heavy physical trainings ^16^. Sources of exogenous antioxidants are Vitamin E, C and also beta-carotene. External antioxidants from food or supplements can help the body to fight an excess of free radicals. In a previous study, proanthocyanidin from grape seed was given to rats for 2 weeks. As a result, it lowered malondialdehyde level and increased the superoxide dismutase and glutathione peroxidase was activated significantly. Furthermore, it reduced fatigue after physical activitie ^17^.

Red-fleshed pitaya ( *Hylocereus polyrhizus*) is a unique fruit with a lot of benefits. The fruit is recently popular among Indonesians and appears to be a natural antioxidant. Several i *n vitro* studies have revealed that red-fleshed pitaya extract has the potential to be an antioxidant ^18^. This research aimed to investigate the antioxidant potential in red-fleshed pitaya extract on HSP70 and cortisol expression in rats that were subjected to strenuous exercise.

## Methods

### Experimental animals

The subjects of this research were 32 Sprague Dawley male rats, aged 3 months, with an average weight of 200 g, were obtained from the Animal Holding Unit of the Pharmacy laboratory, University of Sumatera Utara, Indonesia.

All rats were sustained and maintained in groups (four mice per cage) in experimental animal cages of the Pharmacy laboratory, University of Sumatera Utara, Indonesia. The cage is made of plastic (30 x 20 x 10 cm) and covered with fine wire mesh. The base of the cage is covered with rice husk as thick as 0.5 - 1 cm and replaced every day during the research. The room light was controlled to be exactly at 12 hours light and 12 hours dark cycle, while the temperature 25–27°C and humidity of the room were adjusted to a normal range and fed with standard rat pellets 551, and drink (tap water) was given *ad libitum*.

### Study design

The research applied a laboratory experiment method with random group posttest-only design. The male rats were obtained from the Pharmacy Laboratory, University of North Sumatra. The experimental animals were simple random sampling divided into four groups: Group 1 was subjected to strenuous exercise and treated with distilled water only; Group 2 was subjected to strenuous exercise and treated with dosage 100 mg/kg body weight of red-fleshed pitaya extract; Group 3 was subjected to strenuous exercise and treated with dosage 200 mg/kg body weight of red-fleshed pitaya extract; Group 4 was subjected to strenuous exercise and treated with dosage 300 mg/kg body weight of red-fleshed pitaya extract.

### Red-fleshed pitaya extract

Red-fleshed pitaya fruit, obtained from farmers, in Indonesia, was peeled, washed, cut into small pieces and then dried in a drying cabinet. After that, the fruit was blended using a blender. The fruit extract was isolated through maceration method by using ethanol 96% which has been distilled as much as 10 times the weight of red-fleshed pitaya powder. Red-fleshed pitaya fruit powder in a container had 96% ethanol added to it (ratio 1:7, fruit powder: ethanol), and then was soaked for 3 days then filtered and sealed. The macerates were collected in a container and then processed with rotary evaporator at a temperature of 45°C until the extract was thickened. After that, the same process of were repeated the remaining ethanol 96% for 3 days. The less thickened extract was then evaporated in a water bath until a thick extract was obtained.

100 mg red-fleshed pitaya extract was weighed. Then, it was gently ground using a pestle and mortar. After that, carboxy methyl cellulose (CMC) Na 0.5% solution was slowly added and ground until a homogeneous phase was achieved. Finally, the suspension was added to a 10 mL measuring flask until it reached the mark line. The allocation of red-fleshed pitaya extract, dosage of 100 mg/kg body weight, for instance: weight of 200 g, volume taken: 2 ml extract suspension. Dosage of 200 mg/kg body weight, for instance: weight of 200 g, volume taken: 4 ml extract suspension. Dosage of 300 mg/kg body weight, for instance: weight of 200 g, volume taken: 6 ml extract suspension.

### Experimental procedures

Strenuous exercise given to all rats was a morning swim between 8–9 am for 20 minutes/day, 3 days a week over 21 days. The equipment used in this research was a 10-cm length and 25-cm diameter bath as a pool. Group 1, the rats received distilled water only; Group 2, the rats received 2 ml red-fleshed pitaya extract suspension; Group 3, the rats received 4 ml red-fleshed pitaya extract suspension; Group 4, the rats received 6 ml red-fleshed pitaya extract suspension. Administration of red-fleshed pitaya extract suspension and water was performed orally once daily for 21 days.

Testing for HSP70 and cortisol was conducted two days after the rats had completed a strenuous exercise, 3 days a week over 21 days. During the test, the rats were given a maximum training session by swimming as hard as they could until the rats drowned or showed fatigue symptoms such as the entire body almost dipped into water and limb movements slowed down. After that the rats were sacrificed by placing them in a jar containing cotton which was moistened with 10 ml of chloroform. After that, 2–3 ml blood was taken from the heart. 

Blood samples were collected in micro tubes and centrifuged at 3000 rpm for 15 minutes. The serum was separated and stored at a temperature of 20°C until the analysis process would be carried out. Cortisol was measured with enzyme-linked immune sorbent assay (ELISA); Mouse Cortisol Elisa kit (catalog: E1483Mo, Brand Bioassay TL) and UV spectrophotometry at a wavelength of 450 nm. The HSP70 expression was recorded with ELISA; Mouse HSP70 Elisa Kit (catalog: E0302Mo, Brand Bioassay TL); the absorbance was indicated at 405 nm.

### Statistical methods

Data was analyzed using SPSS 22 for Windows and displayed in tables and diagrams. Normality test was conducted through Shapiro-Wilk test (P > 0.05) in order to determine the average of normal distribution of sample data which is presented as mean ± SD. The result of the normality test was used for next analysis; parametric analysis was used for normal distribution, otherwise non-parametric analysis was used. The ANOVA statistical analysis was performed to indicate the effects of treatments for each group. If the significant result is obtained, then the procedure is followed by Least Significance Difference or Bonferroni tests.

### Ethical approval

The research was performed on the animal subjects were in according with the ethical standards by the Animal Research Ethics Committees/AREC, Faculty of Mathematics and Natural Sciences University of Sumatera Utara, Indonesia (approval number 0011/KEPH-FMIPA/2018).

All efforts were made to reduce any suffering of the rats was during the experiments by following careful procedures and also by anaesthetizing the animal prior to scarifice to prevent experiencing any pain.

## Results

A normality test indicated that the data are normally distributed ( Table 1). HSP70 expression was decreased across all groups (69.57 vs 46.04 vs 31.47 vs 27.65 pg/mL). Group 4 had the lowest expression compared with the other groups. This research reveals a significant decrease in HSP70 expression (p=0.000) between the groups ( Table 2).

**Table 1.** Normality test for heat shock protein 70 (HSP70) and cortisol expression.

Parameter	Group	Normality test	
		statistic	p-value
HSP70	Group 1	0.969	0.888
	Group 2	0.907	0.332
	Group 3	0.922	0.449
	Group 4	0.875	0.168
Cortisol	Group 1	0.923	0.451
	Group 2	0.962	0.833
	Group 3	0.939	0.603
	Group 4	0.900	0.290

Shapiro-wilk test, P>0.05

Cortisol expression was also decreased across all groups (119.02 vs 86.11 vs 62.94 vs 40.86 pg/mL). Group 4 had the lowest expression compared with the other groups. An ANOVA test revealed a significant decrease in cortisol expression (p=0.000) between the groups ( Table 2).

**Table 2.** Average heat-shock protein (HSP70) and cortisol expression in rats who were subjected to strenuous exercise and treated with distilled water (group 1), and 100 mg/ kg body weight (group 2), 200 mg/ kg body weight (group 3) and 300 mg/ kg body weight (group 4) red-fleshed pitaya extract, respectively.

Parameter	Group 1 (n=8)	Group 2 (n=8)	Group 3 (n=8)	Group 4 (n=8)	p-value
HSP70 (pg/ml)	69.57 ± 7.27	46.04 ± 6.45	31.47 ± 1.24	27.65 ± 1.72	0.000*
Cortisol (pg/ml)	119.02 ± 5.56	86.11 ± 11.40	62.94 ± 4.61	40.86 ± 10.94	0.000*

The mean ± SD HSP70 and cortisol expression in shown

**Figure 1.**
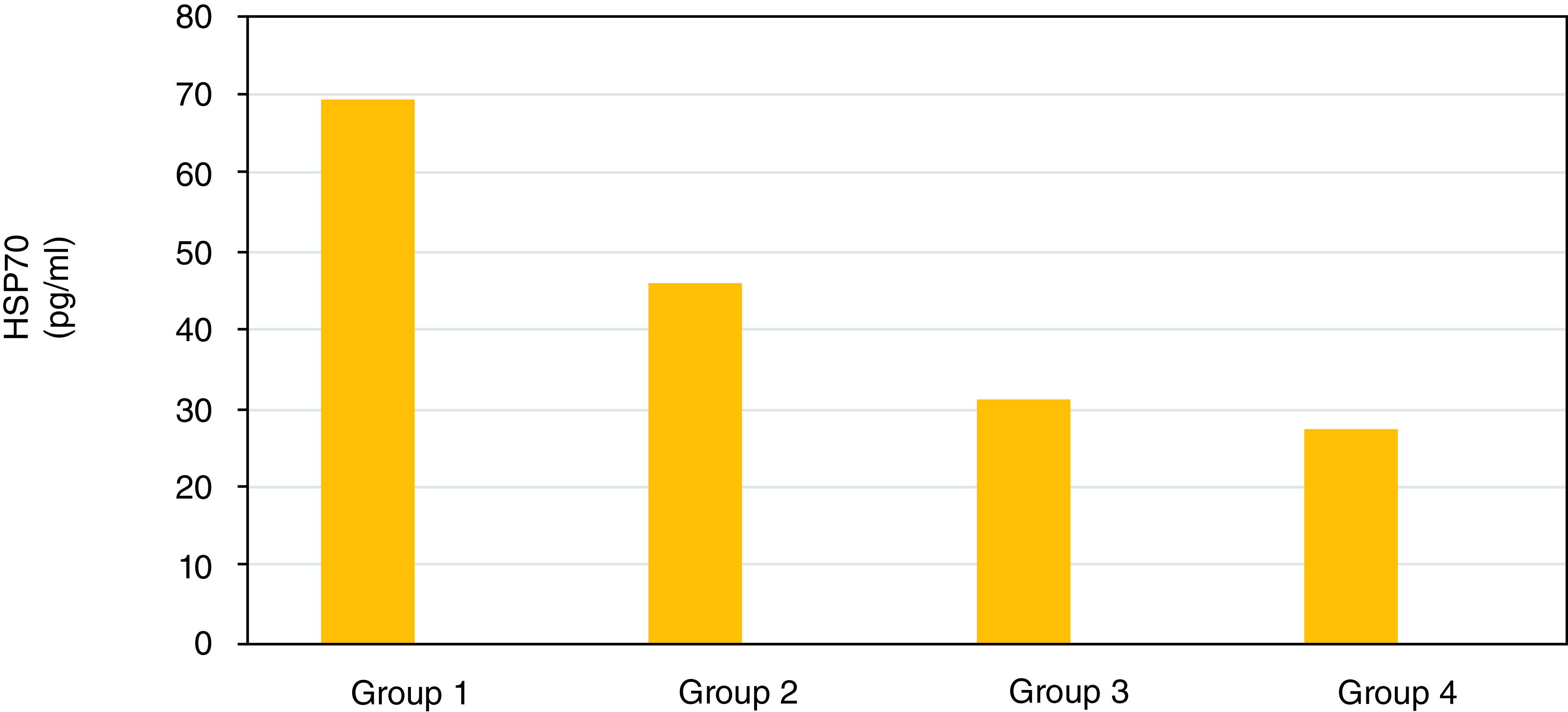
Effect of red-fleshed pitaya extract on HSP70 expression in rats that were given strenuous exercise.

Figure 1 indicates a significant difference in the average decrease of HSP70 expression between group 4 and either group 1 or 2 (p=0.000). However, a significant difference between group 4 and group 3 (p=0.813) was not found. This means that group 4, with strenuous exercise and given 300 mg/kg body weight red-fleshed pitaya extract was indicated to be more effective for reducing HSP70 expression compared to group 1 and 2, with no big variance from group 3.

**Figure 2.**
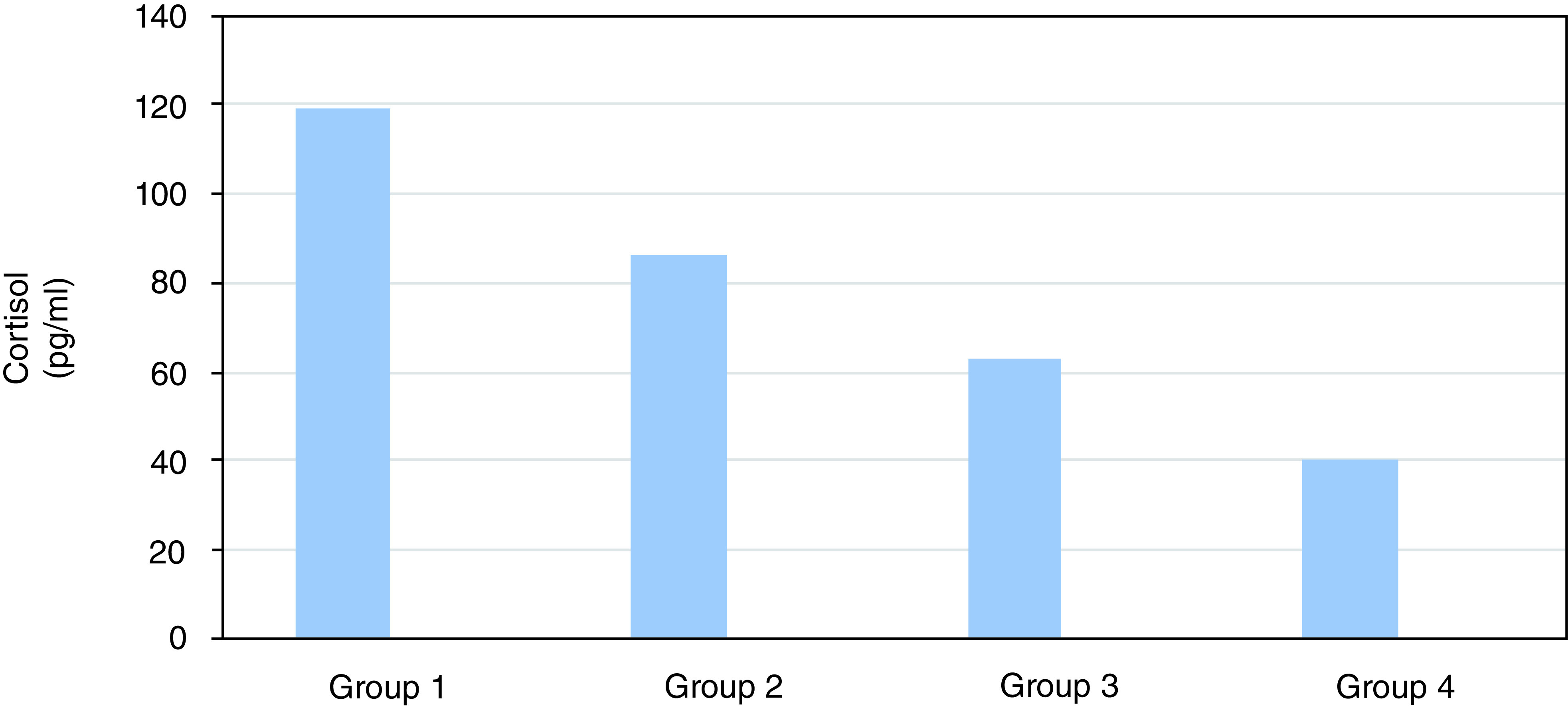
Effect of red-fleshed pitaya extract on cortisol in rats that were given strenuous exercise.

Figure 2 confirms that a significant difference is revealed in the average decrease of cortisol expression between groups 4 and 1 (p=0.000), 2 (p=0.000), and 3 (p=0.000). It can be concluded that group 4 with strenuous exercise and 300 mg/kg body weight red-fleshed pitaya extract was more effective in reducing cortisol expression compared to the other groups.

## Discussion

Based on the results of this research, it is found that strenuous exercise combined with daily red-fleshed pitaya extract consumption contributes to a declining expression of HSP70 and cortisol. The dosage of 300 mg/kg body weight red-fleshed pitaya extract was found to be the optimum amount in decreasing cortisol compared to 100 and 200 mg/kg body weight dosage. However, for both 300 mg/kg body weight and 200 mg/kg body weight dosage of red-fleshed pitaya extract could not provide any difference to HSP70 expression. Therefore, the red-fleshed pitaya extract can be categorized as a potential exogenous antioxidant to eliminate free radicals formed during strenuous exercise.

Free radicals are an element that possesses one or more unpaired electrons in its outermost orbital. Consequently, it is very reactive to cells or cell components in its surroundings. Commonly, a reactive element finds its pair by attacking and binding with adjacent electrons. Then, if this element reacts with another radical element, a new radical element will be formed. This will consistently continue to occur leading to an unavoidable chain reaction ^19^. During strenuous exercise, oxygen consumption rises by 20 times. The excess oxygen triggers the formation of free radicals with electrons released from the respiratory chain. Free radicals production from activity, especially superoxides, increases in the mitochondria ^20^. When an imbalance happens due to the excess of free radicals, oxidative stress occurs and damages the DNA. Moreover, proteins will lose their structure and function like enzymes and membrane receptor. Also, there will be damage in the lipid bilayer structure ^21^.

Strenuous exercise triggers oxidative stress to occur, therefore, HSP70 expression will elevate, resulting in a decrease of endogenous antioxidant activity. HSP70 is an important protein molecule for cell healing and preventing homeostasis, and also increased expression as a cyto-protective effect ^22^. HSP70 is induced strongly due to oxidative stress as cyto-protective to prevent oxidative damage and heal broken proteins ^23^. The increase of HSP70 expession is aimed to balance between ischemic condition, high temperature and increased production of free radical ^22^. In this research, it is found that HSP70 expression tends to decrease in a group with strenuous exercise combined with red-fleshed pitaya extract.

The red-fleshed pitaya extract combined with strenuous exercise is proven to be able to inhibit the increasing HSP70 expression. This occured due to the potential of red-fleshed pitaya as an antioxidant that is able to balance the increasing amount of free radicals formed and impairs HSP70 synthesis induced by strenuous exercise ^24^. Therefore, HSP70 expession is lower compared to the groups that are not given by red-fleshed pitaya extract. The results of this research is in accordance to a research condcuted by Petiz *et al.* (2017) which discovered that Vitamin A combined with high intensity acivities could prevent tissue damage and reduce endogenous antioxidant defense regulation in rats, which also suppressed HSP70 expression ^25^. Other researches have also revealed that antioxidant supplementation is proven to be effective to slow down the HSP70 synthesis caused by high intensity exercise ^24^.

Physical training combined with low to medium dosage of red-fleshed pitaya extract were measured based on the ability and lead to immunomodulation effect that affect the body immune system and protect the body from cell damages, resulting in an effective condition to reduce the oxidative stress ^26^. Stress resulted from physical activity like oxidative stress is responded by hypothalamus to secrete corticotrophin realizing hormone (CRH) which then delivers a message to pituitary anterior. The pituitary produced adrenocorticotropic hormone (ACTH) which is useful to activate or affect adrenal cortex where cortisol hormone is secreted. Cortisol contributes a massive influence to immune responses ^14^ as a sign that the body is suffering from oxidative stress ^27^. In this research, cortisol hormone level decreased in the group with strenuous exercise combined with red-fleshed pitaya extract.

This occurs since the red-fleshed pitaya extract is an effective antioxidant to reduce the risk of oxidative stress and is able to decrease the secretion of cortisol by reducing ACTH secretion in the hypothalamus and CRH in the pituitary gland ^28^.

## Conclusion

Based on this research, it can be concluded that red-fleshed pitaya extract has the potential to be antioxidant with its anthocyanin content, and is able to eliminate oxidative stress due to strenuous exercise. It can be observed by the decreased pattern of HSP70 and cortisol expression in the strenuous exercise combined with red-fleshed pitaya extract group. Furthermore, the optimum result was shown in the dosage of 300 mg/kg body weight red-fleshed pitaya extract.

## Data availability

### Underlying data

Open Science Framework: The effect of red-fleshed pitaya ( *Hylocereus polyrhizus*) on heat shock protein 70 and cortisol expression in strenuous exercise induced rats, https://doi.org/10.17605/OSF.IO/MGX4K ^29^

Data are available under the terms of the Creative Commons Zero “No rights reserved” data waiver (CC0 1.0 Public domain dedication).
