# Supplementary material for: The effect of red-fleshed pitaya ( Hylocereus polyrhizus) on heat shock protein 70 and cortisol expression in strenuous exercise induced rats
Source: F1000Res. 2021 Sep 29;8:130. Originally published 2019 Jan 30. [Version 2] doi: 10.12688/f1000research.17533.2 (PMC8517728; doi:10.12688/f1000research.17533.2)
Supplement: Peer review history

# Peer review: Reviewer response for version 2

**DOI:** 10.5256/f1000research.27866.r95720

**Published:** 2021-10-14

**Authors:** Yusni Yusni

**Affiliations:** 1 Department of Physiology, Syiah Kuala University, Banda Aceh, Indonesia

I have read the details of the author's revision. I decide that I agree with all revisions made by the author. I think this article deserves to be indexed.

Is the work clearly and accurately presented and does it cite the current literature?

Yes

If applicable, is the statistical analysis and its interpretation appropriate?

Yes

Are all the source data underlying the results available to ensure full reproducibility?

Yes

Is the study design appropriate and is the work technically sound?

Yes

Are the conclusions drawn adequately supported by the results?

Yes

Are sufficient details of methods and analysis provided to allow replication by others?

Yes

Reviewer Expertise:

Medical Physiology: exercise physiology; endocrine and metabolic physiology

I confirm that I have read this submission and believe that I have an appropriate level of expertise to confirm that it is of an acceptable scientific standard.

# Peer review: Reviewer response for version 1

**DOI:** 10.5256/f1000research.19175.r59870

**Published:** 2020-03-04

**Authors:** Mohd Adzim Khalili Rohin

**Affiliations:** 1 Faculty of Health Sciences, Sultan Zainal Abidin University (UniSZA), Kuala Nerus, Malaysia

1. It is an interesting manuscript. However, there are several grammatical issues and some typos that you should check very carefully.
2. My main concern about your manuscript is that you have not mentioned any strengths or limitations in your study.
3. The small number of rats per group is indeed a limitation and should be mentioned and taken into consideration before making conclusions.
4. This is a prospective study on the effect of red-fleshed pitaya ( *Hylocereus polyrhizus*) on heat shock protein 70 and cortisol expression in strenuous exercise-induced rats.
5. The conclusion is weak in my opinion.
6. The study could be considered more as a pilot study than a proper study; however the results are interesting and should be indexed.

Is the work clearly and accurately presented and does it cite the current literature?

Yes

If applicable, is the statistical analysis and its interpretation appropriate?

Yes

Are all the source data underlying the results available to ensure full reproducibility?

Yes

Is the study design appropriate and is the work technically sound?

Yes

Are the conclusions drawn adequately supported by the results?

Yes

Are sufficient details of methods and analysis provided to allow replication by others?

Yes

Reviewer Expertise:

Functional Food & Nutraceuticals

I confirm that I have read this submission and believe that I have an appropriate level of expertise to confirm that it is of an acceptable scientific standard.

# Peer review: Reviewer response for version 1

**DOI:** 10.5256/f1000research.19175.r55888

**Published:** 2019-11-08

**Authors:** Yusni Yusni

**Affiliations:** 1 Department of Physiology, Syiah Kuala University, Banda Aceh, Indonesia

**Introduction:**

1. Please explain why red-fleshed pitaya is thought to act as antioxidants that will affect the heat shock protein 70 and the expression of cortisol: include theoretical and other studies that support.
2. The novelty of the article must be clearly explained in the introduction.

**Methods:**

1. Why use 3 doses, whereas the purpose of the study did not assess the effective dose? In the conclusion, also mention the effective dose.
2. Explain the results of the identification of the chemical content of red-fleshed pitaya and also the results of herbarium analysis because there is a possibility that Indonesia's geographical differences will affect its chemical content.
3. Explain the time of blood-sampling because cortisol production is influenced by circadian.

**Discussion:**

1. It is important to explain the role or mechanism of action of red-fleshed pitaya on heat shock protein 70 and cortisol expression.

**Reference:**

1. Use the latest references (from the last 5-10 years).

**Note: **There are some grammatical errors, you should use the proof-read service.

Is the work clearly and accurately presented and does it cite the current literature?

Yes

If applicable, is the statistical analysis and its interpretation appropriate?

Yes

Are all the source data underlying the results available to ensure full reproducibility?

Yes

Is the study design appropriate and is the work technically sound?

Yes

Are the conclusions drawn adequately supported by the results?

Yes

Are sufficient details of methods and analysis provided to allow replication by others?

Yes

Reviewer Expertise:

Medical Physiology: exercise physiology; endocrine and metabolic physiology

I confirm that I have read this submission and believe that I have an appropriate level of expertise to confirm that it is of an acceptable scientific standard, however I have significant reservations, as outlined above.

# Response

**Published:** 2019-11-26

**Authors:** Novita Sari Harahap

**Affiliations:** IDI, Indonesia

**Introduction:**

1. Please explain why red-fleshed pitaya is thought to act as antioxidants that will affect the heat shock protein 70 and the expression of cortisol: include theoretical and other studies that support.

*Red fleshed pitaya has antioxidant activity. Red Dragon Fruit contains powerful antioxidants because it consists of various antioxidants, namely flavonoids, vitamin E, vitamin C (Lianiwati, 2011). The results of several studies stated the red dragon fruit (Hylocereus polyrhizus) has an anthocyanin content (Kanner et al., 2001) around 26,457 ppm. Dragon fruit contains flavonoids (Damayanti, 2016). Anthocyanin is a pigment that is classified as a type of water-soluble flavonoids. Anthocyanins are anthocyanidin and glucose compounds in organic acids (Shipp and Abdel Aal, 2010). Anthocyanins are believed to have a very good antioxidant effect. A study conducted at the University of Michigan in America showed that anthocyanin can destroy free radicals, more effectively than vitamin E which has been known as a powerful antioxidant. Other studies in the United States prove that anthocyanin is the most powerful antioxidant among other flavonoid classes. The content of anthocyanin is believed to inhibit various free radicals such as superoxide radicals and hydrogen peroxide. Anthocyanins and various forms of their derivatives can inhibit various oxidation reactions by various mechanisms (Astawan and Kasih, 2008). The results of previous studies ripe dragon fruit contains many organic acids (Stinzing, et al., 2004), protein (Le Bellec, et al., 2006), minerals such as potassium, magnesium, calcium and iron and vitamin C (Cal et al, 2003).*

**

*Increased RONS (Reactive Oxygen and Nitrogen Species) production during exercise has potential negative effects, transient increases in RONS seem to be a trigger for many exercise-induced adaptations in skeletal muscle (Powers et al. 2010). Exercise can stimulate an increased concentration of HSP70 in muscles and plasma.*

**

*Oxidative stress due to exercise can cause physiological functional changes because physical exercise is considered as a stimulus received by the hypothalamus, then the hypothalamus signals to the HPA axis, the HPA axis responds and gives positive and negative responses to the body (Guilliam & Edward, 2010). Then stimulates the hypothalamus and causes secretion of the hormone corticotrophin-releasing hormone (CRH), which stimulates the hypothalamus for ACTH secretion. Increased ACTH secretion, causes increased cortisol secretion (Usui et al, 2012).*

**Methods:**

1.  Why use 3 doses, whereas the purpose of the study did not assess the effective dose? In conclusion, also mention the effective dose.

*Giving 3 doses of 100 mg/kg BW, 200 mg/kg BW and 300 mg/kg BW to find out what is the optimal dose to neutralize oxidative stress due to strenuous physical exercise. A dose of 100 mg/kg BW can also reduce oxidative stress but not as well as a dose of 300 mg/kg BW. So it is necessary to experiment with 3 doses, the purpose of the study to find out the potential of Red-fleshed pitaya as an antioxidant and once to know the optimal dose to reduce oxidative stress.*

2. Explain the results of the identification of the chemical content of red-fleshed pitaya and also the results of herbarium analysis because there is a possibility that Indonesia's geographical differences will affect its chemical content.

*Red-fleshed pitaya is the cactus fruit of the genera Hyloreceus and Selenicereus. Dragon fruit is very popular and widely planted in Indonesia because it is known by the public as herbal medicine (Evi, et al., 2007). From the results of previous studies, ripe dragon fruit contains many organic acids (Stinzing, et al., 2004), protein (Le Bellec, et al., 2006), minerals such as potassium, magnesium, calcium and iron and vitamin C (Cal et al., 2003).*

3. Explain the time of blood-sampling because cortisol production is influenced by **circadian.**

*Cortisol levels are affected by circadian rhythm. Blood drawn for examination of cortisol is done in the morning because it is a basal state of the body after resting at night. Also besides, our bodies have biological variations according to time, meaning that the analytical levels examined in the morning can give different results if examined in the afternoon. On examination of Cortisol, the levels will increase in the morning and reach the lowest levels in the afternoon. Then the blood draw is done in the morning according to the normal human biological clock, the most efficient and does not result in excessive fatigue.*

**Discussion:**

1. It is important to explain the role or mechanism of action of red-fleshed pitaya on heat shock protein 70 and cortisol expression.

*Research conducted by Rebecca et al (2010) shows that red dragon fruit contains the most polyphenols compared to other species, namely 86.13 ± 17.02 mg in 0.50 g of dried extract of red dragon fruit. Red dragon fruit (Hylocereus polyrhizus) is one of the plants that can be used as a source of antioxidants. Severe physical exercise is associated with increased HSP70 expression due to oxidative stress (Paulsen et al. 2007).*

*Oxidative stress causes damage to cells that will cause stress responses in the form of heat shock response (HSR). HSR is regulated at the level of transcription by a mechanism involving heat shock transcription factor (HSF), especially HSF-1. Increased HSF-1 will help in increasing HSP 70 expression in certain amounts/levels in serum and blood plasma (Baird et al., 2006).*

*Physical activity appears to occur concomitantly with the up-regulation of endogenous antioxidant systems (Powers and Jackson 2008) and heat shock proteins (HSP) in skeletal muscle (Morton et al. 2009b). While the main role of the antioxidant enzymes is to reduce oxidation and prevent oxidative damage, HSPs can prevent and reverse damage to proteins. Intriguingly, the HSPs work with the antioxidant systems, and collectively they have essential roles in cell homeostasis. Up-regulation of these proteins is, therefore, important adaptations for increased protection and recovery capacity in the face of cellular stress and damage induced by high-intensity exercise (Powers and Jackson 2008; Morton et al. 2009b).*

*Antioxidants have been shown to abolish the acute exercise-induced increases in HSP70 protein levels (Khassaf et al. 2003; Jackson et al. 2004).*

*Increased cortisol levels after physical activity depends on the level of fitness, exercise intensity and exercise program [Leite et al., 2011]. Exercise with heavy intensity tends to produce free radicals which can cause the production of the hormone cortisol to increase (Stachowicz, 2016).*

*The provision of red dragon fruit that contains antioxidant polyphenols can reduce the production of free radicals due to exercise thereby reducing cortisol levels.*

# Response

**Published:** 2020-07-04

**Authors:** Novita Sari Harahap

**Affiliations:** IDI, Indonesia

Thank you for this thorough review of the manuscript and your insightful comments that have improved the clarity and quality of the paper. Below there is a point-by-point response to your inquiries.

The difference between this new version and version 1 is that in this new version, we can explain the antioxidant activity contained in red-fleshed pitaya that can affect heat shock protein 70 and the expression of cortisol. Red fleshed pitaya contains powerful antioxidants because it consists of various antioxidants, namely flavonoids, and polyphenol (Chemah et al. 2010; Adnan, 2011). Thus Red fleshed pitaya which has the potential as an antioxidant can reduce ROS and respond to HSP70 expression.

We can also explain that giving 3 doses of 100 mg/kg body weight, 200 mg/kg body weight, and 300 mg/kg body weight to find out what is the effective dose to neutralize oxidative stress due to strenuous physical exercise.  So it is necessary to experiment with 3 doses, in accordance with the purpose of the study to find out the potential of Red-fleshed pitaya as an antioxidant and once to know the effective dose to reduce oxidative stress which is influence on HSP70 and cortisol expression in rats that were subjected strenuous exercise.

Red-fleshed pitaya is the cactus fruit of the genera Hyloreceus and Selenicereus. Red-fleshed pitaya is very popular and widely planted in Indonesia because it is known by the public as herbal medicine. Research by Widyaningsih et al., (2017) report that red-fleshed pitaya as an alternative treatment for anemia in pregnant women. The other studies by Irmayanti and Ardiaria (2016) report that red dragon fruit (Hylocereus Polyhizus) can reduce cholesterol levels in rats. This statement explains that red-fleshed pitaya have also affect difference.
